# Task-evoked simultaneous FDG-PET and fMRI data for measurement of neural metabolism in the human visual cortex

**DOI:** 10.1038/s41597-021-01042-2

**Published:** 2021-10-15

**Authors:** Sharna D. Jamadar, Shenjun Zhong, Alexandra Carey, Richard McIntyre, Phillip G. D. Ward, Alex Fornito, Malin Premaratne, N Jon Shah, Kieran O’Brien, Daniel Stäb, Zhaolin Chen, Gary F. Egan

**Affiliations:** 1 Monash Biomedical Imaging, Monash University, Melbourne, VIC Australia; 2 Australian Research Council Centre of Excellence for Integrative Brain Function, Clayton, Australia; 3 Turner Institute for Brain and Mental Health, Monash University, Clayton, VIC Australia; 4 National Imaging Facility, Clayton, Australia; 5 Department of Medical Imaging, Monash Health, Clayton, VIC Australia; 6 Department of Electrical and Computer Systems Engineering, Monash University, Clayton, VIC Australia; 7 Institute of Neuroscience and Medicine - 4, Forschungszentrum Jülich, Jülich, Germany; 8 MR Research Collaborations, Siemens Healthcare Pty Ltd, Clayton, Australia; 9 Monash Data Futures Institute, Monash University, Clayton, Australia

**Keywords:** Neural circuits, Cognitive neuroscience

## Abstract

Understanding how the living human brain functions requires sophisticated *in vivo* neuroimaging technologies to characterise the complexity of neuroanatomy, neural function, and brain metabolism. Fluorodeoxyglucose positron emission tomography (FDG-PET) studies of human brain function have historically been limited in their capacity to measure dynamic neural activity. Simultaneous [18 F]-FDG-PET and functional magnetic resonance imaging (fMRI) with FDG infusion protocols enable examination of dynamic changes in cerebral glucose metabolism simultaneously with dynamic changes in blood oxygenation. The Monash vis-fPET-fMRI dataset is a simultaneously acquired FDG-fPET/BOLD-fMRI dataset acquired from n = 10 healthy adults (18–49 yrs) whilst they viewed a flickering checkerboard task. The dataset contains both raw (unprocessed) images and source data organized according to the BIDS specification. The source data includes PET listmode, normalization, sinogram and physiology data. Here, the technical feasibility of using opensource frameworks to reconstruct the PET listmode data is demonstrated. The dataset has significant re-use value for the development of new processing pipelines, signal optimisation methods, and to formulate new hypotheses concerning the relationship between neuronal glucose uptake and cerebral haemodynamics.

## Abstract (summary)

**Table:** 

Measurement(s)	brain activity
Technology Type(s)	functional magnetic resonance imaging • FDG-Positron Emission Tomography
Sample Characteristic - Organism	Homo sapiens

Machine-accessible metadata file describing the reported data: 10.6084/m9.figshare.14977851

## Background & Summary

The enhanced abilities of the human brain to plan complex behaviour, make decisions, and process emotional and social contexts comes with heavy energy requirements. Although it accounts for only 2% of the body’s weight, the human brain accounts for 20% of its resting metabolism^1,2^. Oxygen and glucose are the two primary sources of energy in the human brain. The largest proportion of energy consumed by the brain, around 70–80%, occurs during neuronal computation and information processing^3^.

Understanding how the living human brain functions requires sophisticated *in vivo* neuroimaging technologies to characterise the complexity of neuroanatomy, neural function and brain metabolism. The ability to simultaneously image dynamic brain function and metabolic activity in living people was unimaginable just a decade ago. The technological developments that have led to simultaneous positron emission tomography (PET) – magnetic resonance imaging (MR)^4^ has now made it possible to simultaneously measure changes in blood oxygenation and glucose metabolism using simultaneous FDG-PET/BOLD-fMRI ([18 F]-fluorodeoxyglucose PET - blood oxygenation level dependent functional MRI). While BOLD-fMRI provides the capacity to measure changes in blood oxygenation in response to neuronal activity with high spatial resolution and moderate temporal resolution (~2 sec, sub-second with multiband acquisitions), the temporal resolution of traditional static FDG-PET studies is equal to the scan duration (i.e., a snapshot is taken across the scan period, 10–30 mins). As such, FDG-PET studies of human brain function have historically been limited in their capacity to measure the dynamic nature of neural activity.

Recent advances in FDG infusion protocols have made it possible to study dynamic changes in cerebral glucose metabolism simultaneously with dynamic changes in blood oxygenation. In a landmark study, Villien *et al*.^5^ adapted the slow infusion technique^6^ to show dynamic changes in glucose metabolism in response to checkerboard stimulation in the visual cortex with a temporal resolution of 1-min. Subsequent studies using this ‘functional PET’ (fPET) technique have extended these findings to demonstrate simultaneous BOLD and FDG activity in the visual and motor cortices^7–11^, in response to cognitive tasks^12^ and at rest^13,14^. The results of these studies indicate subtle commonalities and differences in patterns of blood oxygenation and glucose metabolism, which are not evident when measuring these responses separately.

Simultaneous FDG-fPET/BOLD-fMRI is a nascent technique which shows substantial promise for understanding the dynamic use of glucose and oxygen during neuronal activity. However, by comparison to BOLD-fMRI and other neuroimaging techniques, the processing pipelines are immature, and have not had the benefit of many years of work validating data preparation and signal detection optimisation. Furthermore, given the difficulty in acquiring these data – requiring access to a molecular MR scanner, access to radioisotopes and hot lab facilities, radiographer and nuclear medicine technologist specialists, as well as specialised MR-compatible equipment for dose delivery^11^ – only a handful of biomedical imaging facilities currently have the capability to acquire this multimodal data.

Here we describe the **Monash vis-fPET-fMRI dataset**, a simultaneous FDG-fPET/BOLD-fMRI dataset acquired from young healthy individuals. The dataset comprises unreconstructed fPET list-mode sinogram data, as well as PET image data reconstructed into 1-min time intervals. The dataset also includes simultaneously acquired reconstructed BOLD contrast MRI image data. The data was acquired using a flickering checkerboard embedded block design^10^, which allows the examination of a task-evoked signal with a known time course in a localised region-of-interest. The design is particularly suitable for development of novel processing and analysis pipelines. Note that this dataset uses *low dose* PET, with an average dose of 93MBq, range 39–105MBq (see Methods). One goal of the PET field has been to improve image quality while reducing the radiation exposure to the patient, two apparently contradictory aims^15^. One way to improve image quality without increasing radiation dose is to apply novel signal processing methods such as machine learning to low-dose PET images^16^. Recent advances in the development of machine learning algorithims for low dose PET have shown promise^16–18^, however additional studies using a diverse range of training sets is required to fully demonstrate the feasibility of low-dose acquisition protocols^15^. The dataset therefore shows promise for re-use in the development of methods to reduce the radiation exposure to patients without sacrificing image quality.

The release of unreconstructed and reconstructed PET data acquired during task-evoked visual cortex activation provides significant re-use value. This data has previously been used to test the effectiveness of the embedded block design to yield fMRI and fPET contrast^10^, to develop data fusion analyses based on independent component analysis (ICA)^19^, and to develop novel PET reconstruction methods based on Bowsher prior^17^. Examples of re-use may include disentangling the glucose metabolic and blood oxygenation level dependent responses to neuronal activity^10,20^, synergistic data reconstruction^17,18,21^ and fusion techniques^19^, novel multimodal attenuation correction procedures^22^, and refinement of fPET-fMRI data processing pipelines^13,23^.

## Methods

All methods were reviewed by the Monash University Human Research Ethics Committee, following the Australian National Statement of Ethical Conduct in Human Research (2007). Administration of ionising radiation was approved by the Monash Health Principal Medical Physicist, following the Australian Radiation Protection and Nuclear Safety Agency Code of Practice (2005). For participants aged over 18-yrs, the annual radiation exposure limit of 5 mSv applies, and the effective dose in this study was 1.9 mSv. Informed consent was obtained from all human subjects in this dataset.

A video methods article demonstrating the data acquisition procedure was reported in Jamadar *et al*.^11^.

### Participants

Participants (n = 10) were aged 18–49 years (mean 29 years), nine female, nine right handed. Data from an additional four participants’ were excluded for the following reasons: occluded cannula during infusion (2), scanner error (1), and one participant fell asleep during the protocol. Participants were screened for diabetes, personal or family history of neurological or neurodegenerative conditions, claustrophobia, MRI safety and nuclear medicine safety. Women were screened for current or possible pregnancy.

Prior to the scan, participants were directed to consume a high protein/low sugar diet for 24hrs, fast for six hours, and drink 2–6 glasses of water. Blood sugar level (BSL) was measured using an Accu-Chek Performa (model NC, Mannheim, Germany). Participants had BSL below 10mML (Table 1; note that BSL was measured but not recorded for one participant).

**Table 1:** Demographic information for each participant.

Subj ID	Age	Sex	Handed-ness	Years of Education	Current/previous neurological condition	Current/previous cardiovascular disease	Current/previous diabetes	Blood sugar level (mML)	Dose (MBq)	Scan duration (mins)	Notes
002	49	female	right	26	Migraine (current)	no	no		39	90	BSL not recorded due to technical error
005	21	female	right	17	no	no	no	5.8	73.3	90	
006	29	female	right	21	no	no	no	4.4	102.42	90	Blood samples not available
007	32	female	right	15	no	no	no	4.6	105	90	
008	48	female	right	20	no	congenital tachycardia, recovered	no	5.4	101	90	Blood samples not available from T4
010	21	male	left	14	no	no	no	5	86.78	95	
012	24	female	left	17	concussion at 8 years old	no	no	4.6	92.079	95	
013	18	female	right	13	no	no	no	5	94.62	95	
014	24	female	right	14	no	no	no	5	101.3	95	
016	19	female	right	19	no	no	no	5	97.87	94	

Demographic information, BSL and administered dose for each of the participants is provided in Table 1.

### Stimuli and tasks

**Fig. 1:**
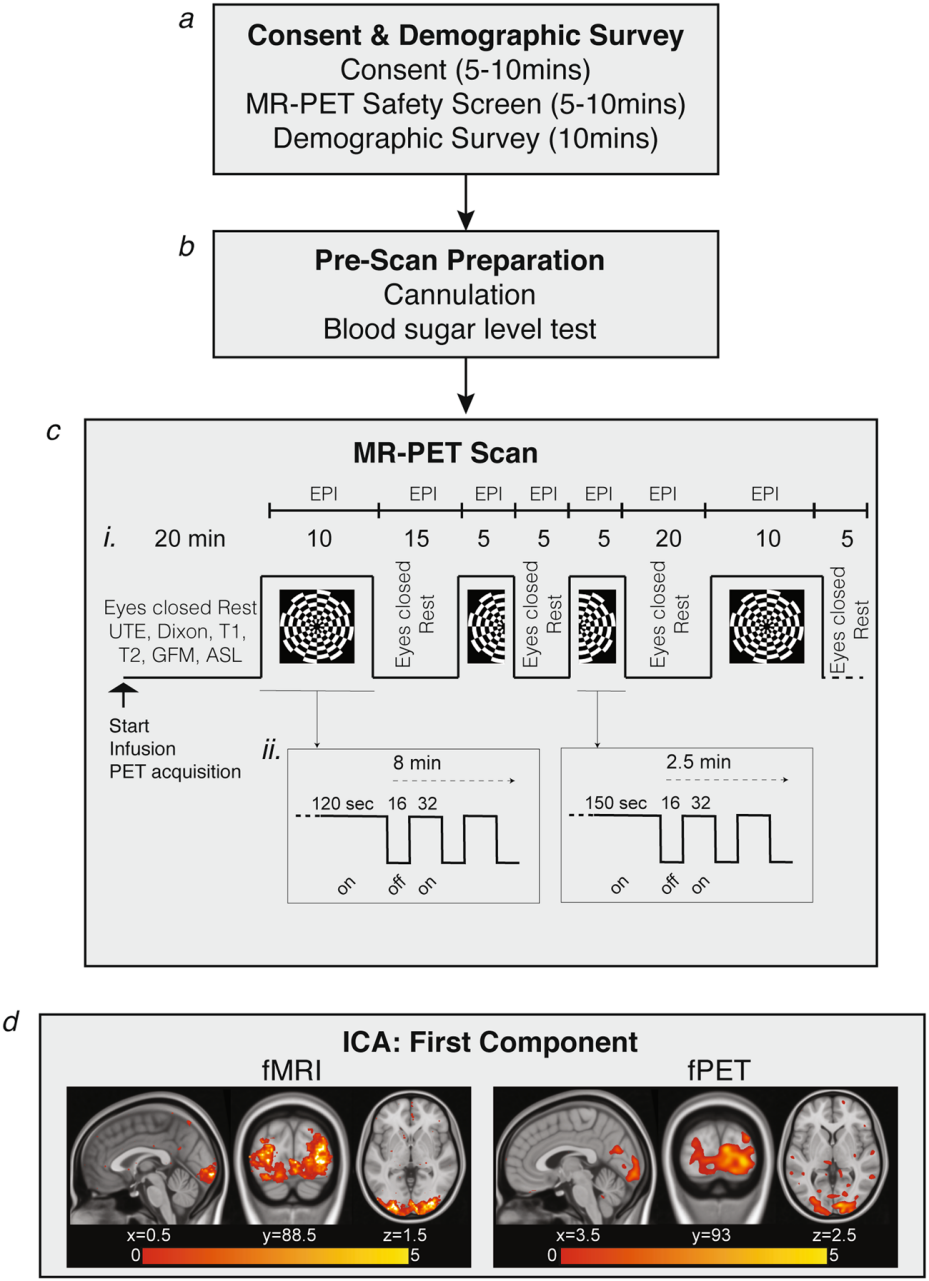
Workflow & Paradigm. (**a**) Participants completed consent, safety screen, and demographic survey in the 30 mins prior to PET-MR scanning. (**b**) Next, participants were prepared for scanning. A cannula was placed in the forearm vein of each arm, then blood sugar level was measured. (**c**) Participants underwent a 90 min PET-MR scan while alternating between eyes closed rest and passive viewing of a visual checkerboard stimulus. i. Timing (minutes) of each component of the paradigm. Infusion and PET acquisition started at time 0. For the first 20-mins non-functional scans were taken while participants rested with their eyes closed. Then, participants completed 10 mins of flickering checkerboard stimulation. Embedded within this 10 min period was a fast on/off design (panel c.ii.), where the checkerboard was shown for 120 sec, and then for the remaining 8 mins, alternated off (16 sec, eye open fixated on cross) and on (32 sec). A 15 min eyes closed rest period then followed, then 5 min left hemifield stimulation, 5 min eyes closed rest, and 5 min right hemifield stimulation. During hemifield stimulation, participants viewed the stimulus with both eyes. Embedded within these 5-mins was a fast on/off design where the hemifield was shown for 150 sec, then alternated off (16 sec, eyes open fixated on cross), and on (32 sec). The protocol concluded with a 20 min eyes closed rest, and a final 10 min full checkerboard block, which had the same parameters as the first full checkerboard block. For participants collected with a 95 min scan, participants were instructed to lay with their eyes closed at the cessation of the final checkerboard block. (**d**) Representative activation results in the visual cortex. A joint independent component analysis (ICA) was conducted on the fMRI and fPET (1-min frames) timeseries. The first joint component is shown for fMRI and fPET. See Jamadar *et al*.^10^ for full report of methods and results of this analysis. Abbreviations: EPI, echo planar imaging; UTE, ultrashort echo time; GFM, gradient field map; ASL, arterial spin labelling, ICA, independent component analysis.

Figure 1 illustrates the workflow for the data acquisition.

An ‘embedded’ block design was developed to produce signal contrast for both fast BOLD-fMRI and slow FDG-fPET measurements (Fig. 1c). The slow on/off task alternation periods (Fig. 1c.i.) were designed to provide FDG-fPET contrast (alternating task and rest over durations of minutes), and fast on/off alternation periods (Fig. 1c.ii.) were embedded in the stimulation periods designed to provide BOLD-fMRI contrast. We made the assumption that the fast on/off periods would alternate at a frequency too high to be detected by the fPET measurements.

Participants rested with eyes closed during the first 20 min block of MRI scans (localiser, T1, etc.). At 20 mins, a 10 min visual stimulation period was presented in an embedded 32/16 sec on/off design. The first 120 sec of the block was a sustained ‘on’ period; we predicted that this period would allow FDG-fPET signal to rise from resting levels. During the ‘on’ periods (i.e., 120 s & 32 s periods), the visual stimulus was a circular checkerboard (size 39 cm, visual angle 9°), presented on a black background. The checkerboard flickered (i.e., alternated black and white fields), at rate of 8 Hz. During the 16 s ‘off’ periods, participants rested with eyes open while viewing a white fixation cross (size 3 cm, visual angle 0°45′) presented on a black background. Following the 10 min stimulation block, a 15 min eyes closed rest period followed, then 5 min of left hemifield stimulation (150 sec on, then 32/16 s on/off), 5 min eyes closed rest, and 5 min of right hemifield stimulation. Note that participants were instructed to open both eyes during hemifield stimulation, and so, activity is likely to be obtained in both hemispheres of the visual cortex during this condition. Following 20 mins of eyes closed rest, a final 10 min full checkerboard (parameters identical to the first 10 min block) was presented. Due to short delays between MR scans, we found that the 90 min duration of the PET scan did not always conclude with the end of the MR sequences. Therefore, we increased the duration of the PET scan for subjects acquired later in the study. PET scan duration for each participant is noted in Table 1. Participants were directed to close their eyes and rest following the cessation of the checkerboard block.

### Procedure

Participants were cannulated in the vein of each forearm with a minimum size 22-gauge cannula, and a 10 mL baseline blood sample was taken. For all participants, the left cannula was used for FDG infusion, and the right cannula was used for blood sampling. Primed extension tubing was connected to the right cannula for blood sampling, via a 3-way tap.

The 90-min simultaneous PET-MR scan was performed using a Siemens Biograph 3 T molecular MR (mMR) scanner (Erlangen, Germany). Participants were positioned supine in the scanner bore with head in a 16ch radiofrequency (RF) head-neck coil. Visual stimuli were viewed via a mirror placed on the RF coil, which reflected a 32-inch Cambridge Research Systems (UK) BOLDscreen MR-compatible LCD screen. [18 F]-fluorodeoxyglucose (FDG; average dose 97.0Mbq) was infused over the course of the scan at a rate of 36 mL/hr using a Caesarea Medical Electronics (Israel) BodyGuard 323 MR-compatible infusion pump. One participant received a dose of 39MBq due to technical error (Table 1). Infusion onset was locked to the onset of the PET scan (see PET-MR Protocol, below).

Plasma radioactivity levels were manually obtained throughout the duration of the scan. At 10-min post-infusion onset, a 10 mL blood sample was taken from the right forearm using a vacutainer; the time of the 5 mL mark was noted for subsequent decay correction. Subsequent blood samples were taken at 10 min intervals for a total of ten samples for the duration of the scan. The cannula line was flushed with 10 mL of saline after every sample to minimise line clotting. As noted in Table 1, blood samples from one subject were unable to be obtained, and samples for one additional subject were unable to be obtained after timepoint 4, due to difficulties drawing blood.

### PET-MR Protocol

PET data was acquired in list-mode. PET data acquisition and infusion of FDG started with the ultrashort TE (UTE) MRI for PET attenuation correction. Non-functional MRI scans were acquired in the first 20-mins, and included T1 3D MPRAGE (TA = 7.01 mins, TR = 1640 ms, TE = 2.34 ms, flip angle = 8°, FOV = 256 × 256 mm^2^, voxel size = 1 × 1 × 1 mm^3^, 176 slices; sagittal acquisition), T2 FLAIR (TA = 5.52 mins), gradient field map (TA = 1.02 mins, TR = 466 ms, TE_1_ = 4.92 ms, TE_2_ = 7.38 ms, voxel size = 3 × 3 × 3mm^3^, FOV = 190 mm, 44 slices), and ASL (TA = 3.52 min, data not released). For the remainder of the scan, seven consecutive blocks of T2*-weighted echo planar images (EPIs) were acquired (TR = 2450 ms, TE = 30 ms, FOV = 190 mm, 3 × 3 × 3 mm^3^ voxels, 44 slices, ascending interleaved axial acquisition). The duration of each EPI block was determined by the stimulus block duration (Fig. 1a): block 1 full checkerboard (TA = 10.02 min, 242 volumes), block 2 eyes closed (TA = 15.01 min, 364 volumes), block 3 half checkerboard (TA = 5.05 min, 121 volumes), block 4 eyes closed (TA = 5.05 min, 121 volumes), block 5 half checkerboard (TA = 5.05 min, 121 volumes), block 6 eyes closed (TA = 20.02, 487 volumes), block 7 full checkerboard (TA = 10.02 min, 242 volumes).

## Data Records

Online-only Table 1 defines the data available for each subject on OpenNeuro. The dataset containing the demographic and anthropometric data, T2* EPI fMR images, PET images reconstructed in 1-min bins, unreconstructed PET list-mode data, T1 structural images and gradient field maps is freely available in BIDS format^24^ from the OpenNeuro repository (http://openneuro.org) with the accession number ds003382 (10.18112/openneuro.ds003382.v1.0.0)^25^.

Participants.tsv is a text file reporting the demographic and anthropometric data for each subject ordered by subject ID. Plasma_radioactivity.tsv is a text file reporting the plasma radioactivity counts and measurement times for each subject, ordered by subject ID.

The Monash vis-fMPRI-fPET dataset contains both raw (unprocessed) images and source data (i.e. unconstructed PET listmode data). Both are organized in sub-directories that are corresponding to the subjects, according to the BIDS specification. For each subject, T1-weighted MPRAGE images, fMRI images and gradient field maps are in the anat (anatomical data), func (functional data) and fmap (field map) sub-directories respectively, along with metadata in the json sidecar. Dixon and UTE scans are also added to the dataset for reconstructing the PET source data into images, which are organized in the ‘dixon’ and ‘ute’ sub-directories. At least two UTE echos are included (sub-*/ute/*ute.nii.gz), and the computed attenuation correction maps are also shared (sub-*/ute/*ute-umap.nii.gz). The UTE sequence parameters are included in the json sidecar, and can be found under the ‘global’ tag, where the values of ‘EchoNumbers’ are corresponding to the first and second UTE echoes. MR coil attenuation has been corrected during PET image reconstruction, however the attenuation from the mirror used for visual stimulation was not corrected due to practical limitations. Although there is not currently a BIDS-compliant format, the same structure is followed with a json sidecar along with each of the image data. Both static and dynamic reconstructed PET images are contained in the ‘pet’ directory. The static PET images (1.5 zooming) are reconstructed, using the Ordered Subset Expectation Maximization (OSEM) (Burgos *et al*., 2014) algorithm with 21 subsets and 3 sub-iterations, and smoothed with 2 mm FWHM Gaussian spatial filter, are stored as sub-*/pet/*static*.nii.gz files The dynamic reconstruction was performed for each 1-min acquisition time interval to produce 90–95 mins of sequential fPET images with a 4 mm Gaussian spatial filter applied. The images are store in the same directory as sub-*/pet/*dynamic_1min-ac.nii.gz files. Following the current working copy of the proposed BIDS Extension for PET (BEP009), blood data (i.e. discrete plasma measurements of radioactivity) are also included in the ‘pet’ directory, which report the plasma radioactivity counts and measurement times for the subject. Data in sub-*/dixon, sub-*/ute and sub-*/pet are ignored in the BIDS validation process, as they are not officially supported by the current BIDS specification.

The ‘sourcedata’ directory contains the raw, non-reconstructed PET source data that was directly exported from Siemens scanner console. The source data includes PET listmode data, normalization data, sinogram data and physiology data. The raw PET data are in the form of a file pair (one DICOM header and one raw binary file) with the two paired files having the same file name but different filename extensions (.dcm for the DICOM file and.bf for raw binary file). A json metadata sidecar file was added for each subject’s raw dataset, similar to the other image types officially supported by BIDS specification. The blood data containing the blood plasma measurements is included identically as for the reconstructed PET image data. The ‘sourcedata’ directory is also excluded in the BIDS validation process.

To prepare the BIDS dataset, the open source conversion tool, Heudiconv (https://github.com/nipy/heudiconv, version 0.8.0) was used to organize the imaging data into structured directory layouts, and the Dcm2niix convertor (https://github.com/rordenlab/dcm2niix, version 1.0.20200427) was used to convert the image data from DICOM to NifTI format. Customized scripts that were written to (i) remove personal identifiable information (PII) from the raw PET dicom header; (ii) add custom json sidecar files to the PET raw data and reconstructed image data; (iii) generate plasma radioactivity files; are available on a github repository (https://github.com/szho42). Defacing was applied to T1-weighted images, Dixon and UTE images, using the tool, pydeface (https://github.com/poldracklab/pydeface, version 2.0.0). The original umaps are shared, as defaced umaps will impact on the reconstruction results, due to losing bone information. The uMaps contain little facing information, which does not violate the OpenNeuro privacy policy. The reconstructed PET images and PET raw data were not defaced as the subjects are not able to be visually identified from the PET images. The scripts used in the BIDS dataset conversion are publicly available (https://github.com/szho42/bids-pet-conv, version 2.0.1).

## Technical Validation

Whilst the Monash vis-fPET-fMRI dataset consists of simultaneous FDG-fPET and BOLD-fMRI datasets, this section primarily focuses on the validation of the fPET raw data as similar fMRI image data has previously been reported^10,13^. The unreconstructed PET listmode data is only useful as a publicly accessible dataset if tools are available for offline binning of the listmode data into sinogram data, and reconstruction of the sinogram data into PET images. Proprietary tools exist to perform offline image reconstructions but these are not publicly available. Technical validation of the images reconstructed using the open source reconstruction frameworks compared to the proprietary tools is required to facilitate meaningful sharing of the PET listmode data. However, the current limitations of the open source frameworks do not enable a direct quantitative comparison between the reconstruction methods. Nevertheless, a qualitative comparison has been undertaken as described below.

### PET Raw Data Image Reconstruction with SIRF

The PET listmode data was reconstructed using the SIRF^21^ framework (with STIR^26^ as the backend, https://github.com/UCL/STIR, version 4.0.2) to demonstrate the technical feasibility of reconstructing the listmode data using open source PET reconstruction tools. The process required use of several open source tools including: (i) pet-rd-tools (https://github.com/UCL/pet-rd-tools, version 2.0.1) for converting the PET raw data from the Siemens-specific format to Interfile format used by the STIR reconstruction toolkit; (ii) the STIR reconstruction toolkit (https://github.com/UCL/STIR) that is a framework for iterative image reconstruction for PET and SPECT raw data; and (iii) the SIRF framework (https://github.com/SyneRBI/SIRF, version 2.2.0) that provides a user-friendly interface to write scripts in Python and Matlab. The SIRF built-in template image for ‘Siemens mMR’ was used, with a span of 11, maximal ring difference of 60 and mashing factor of 2. To reconstruct the fPET raw data dynamically, flexible binning patterns were customized in SIRF by setting specific listmode date start times and the time interval for conversion of the listmode data to sinogram data. In some cases, the first time tag is not zero (which is known as a STIR issue in the current version), in which a small threshold was set to identify the true start time. The corresponding sinogram was generated with the estimated random coincidences map, which were fed into the iterative methods for reconstruction. In our case, UTE-based attenuation maps were used for attenuation correction, which was converted by pet-rd-tool with the orientation of RAS. The acquisition sensitivity model was set by chaining the normalization and attenuation map, and background term was set by combining the randoms map and the estimated scatter map. The Ordered Subsets Maximum A Posteriori One Step Late algorithm (OSMAPOSL) was used to iteratively reconstruct the PET images, using the Poisson log-likelihood objective function. The SIRF reconstruction uses 21 subsets which is identical to the setting on the vendor console, however 40 sub-iterations are applied on the SIRF reconstruction pipeline for achieving reasonable quality images. A 4 mm FWHM Gaussian spatial filter was applied to the PET images after reconstruction. To fairly compare the results, we also reconstruct images on Siemens console, with the same setting, by only enabling 4 mm Gaussian smoothing for both static and dynamic reconstructions.

### Qualitative comparison between the reconstructed PET images

**Fig. 2:**
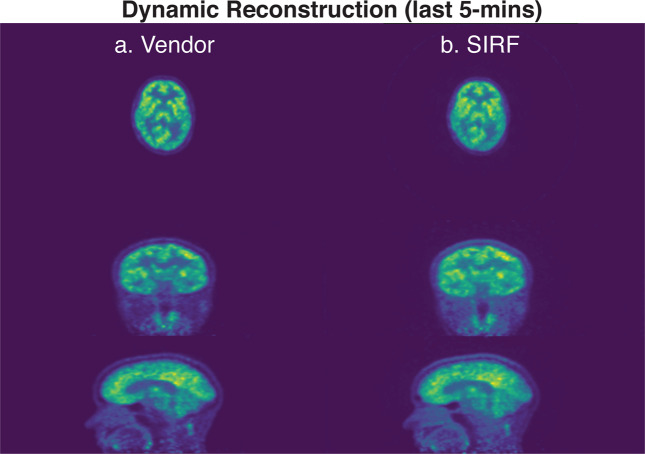
Example of a dynamic reconstructed PET image for subject 005 using the last 5-min of the acquisition data. (**a**) The reconstructed PET images using the vendor reconstruction pipeline. (**b**) The reconstructed PET images using the SIRF^21^ open source framework with random, scatter and attenuation correction applied.

An example of the dynamically reconstructed PET images for 5-minute of the sinogram data are shown in Fig. 2. The reconstructed PET image using (a) the Siemens reconstruction pipeline on the scanner console and (b) the open source framework, SIRF with scatter and UTE-based attenuation correction applied are shown in Fig. 2(a,b) respectively. Both images were reconstructed using the last 5-minute of list mode data for subject 005 in the dataset. It can be seen from Fig. 2 that the listmode data can be reconstructed using open source tools like SIRF, and have very similar contrasts, compared to the images from the Vendor console in a dynamic setup. It is worth mentioning that the image quality using SIRF is similar but still different from the vendor’s reconstruction pipeline. The functionalities in STIR/SIRF are fast developing and we expect further improvements in later releases. Further quantitative validation of the open source PET reconstruction tools will be required as the STIR/SIRF framework is further developed.

**Fig. 3:**
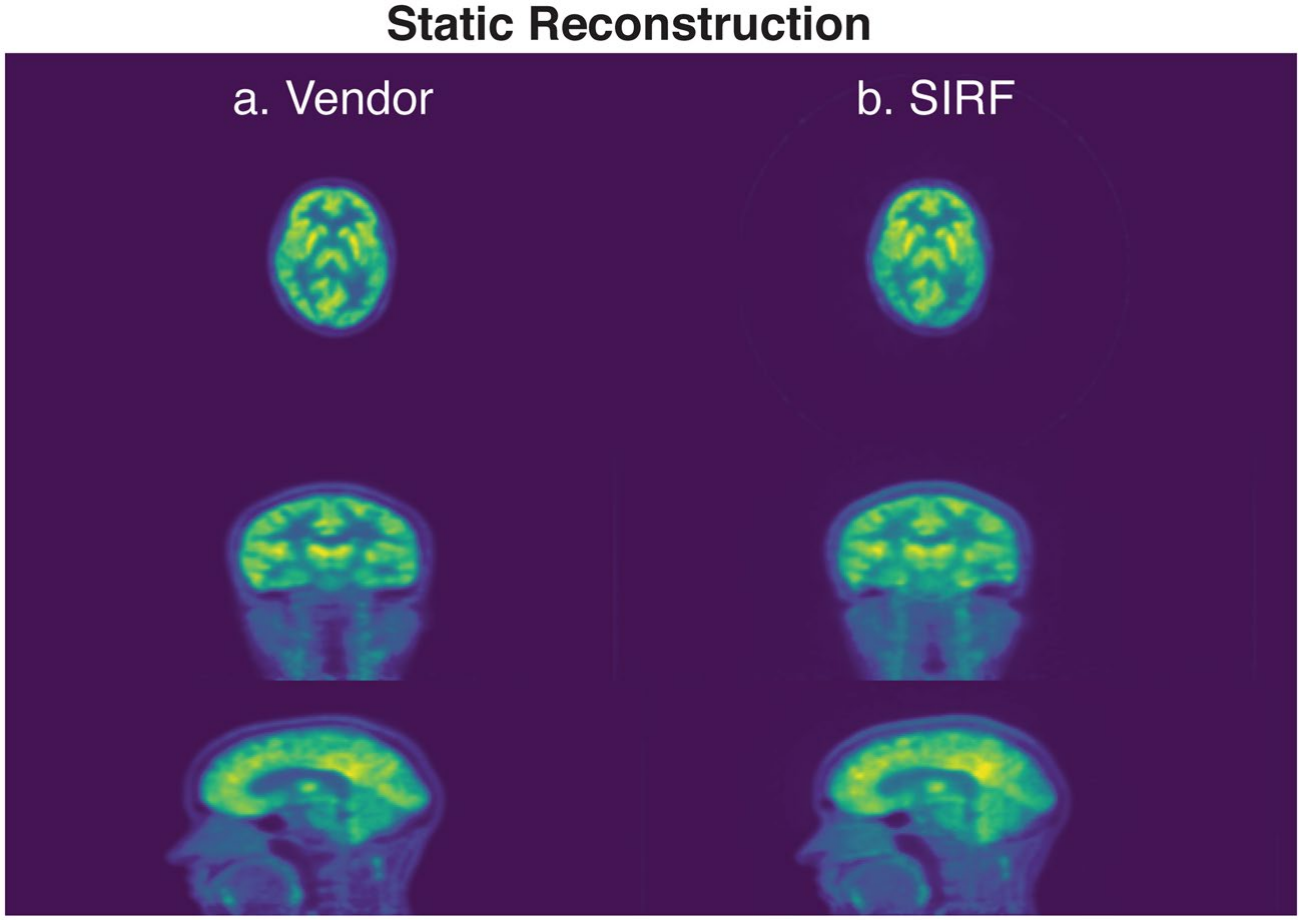
The static reconstructed PET images for subject 005, using (**a**) vendor reconstruction pipeline and (**b**) the SIRF^21^ open source frame with random, scatter and attenuation corrections applied.

For reference, the static PET image reconstructed for one subject (subject 005 using the SIRF opensource framework with random, scatter and UTE-based attenuation correction applied) using the entire listmode acquisition is shown in Fig. 3. As expected, the image demonstrates significantly higher signal to noise in comparison to the PET images from the last 5-min of the acquisition as shown in Fig. 2, as well as superior anatomical localisation of the FDG signal. The scripts used to reconstruct the dynamic and static PET images are publicly available at (https://github.com/szho42/pet-image-recon).

In summary, the STIR/SIRF reconstructed PET images demonstrate that the PET list mode data can be successfully reconstructed, but further validation of the open source reconstruction framework still remains to be undertaken. Future validation work could for example involve reconstruction of the PET list mode data with smaller time intervals (e.g. 30secs) for comparison with comparable images reconstructed using the proprietary Siemens framework. This would have the advantage of increasing the temporal resolution of the fPET image dataset that could potentially increase the sensitivity for the functional PET method to detect visually evoked brain activity.

## Usage Notes

With the release of unprocessed fMR image data and unreconstructed PET list mode data, the Monash vis-fPET-fMRI dataset^1^ has significant re-use value. The primary motivation for the public release of the dataset is to facilitate the development of new processing pipelines, signal optimisation methods, and stimulate new hypotheses concerning the relationship between glucose uptake and cerebral haemodynamics in the human brain. The task-related design is particularly powerful, as the visual stimulation task robustly activates a well localised region-of-interest of visual cortex with a known time-course. The experimental design facilitates functional PET imaging methods development and evaluation of statistical analytical techniques for fPET data^23^. Some examples of re-use would be to explicitly test whether the 5 min on/off hemifield alternation is sufficient to distinguish between stimulation and rest period in the fPET data; or alternatively whether the fast 32/16 sec on/off in the checkerboard stimulation could be differentiated (Fig. 1). Furthermore, the release of unreconstructed PET list-mode data is particularly significant. We believe that this dataset represents the first PET list-mode dataset available through the OpenNeuro platform, and probably the first publicly available list-mode dataset simultaneously acquired with BOLD-fMRI data.

Access to unreconstructed PET list-mode data facilitates the development of novel image reconstruction^17,18^ and attenuation correction methods^22^, including synergistic PET-MR image reconstruction^21^. For researchers interested in standard reconstructions with differing parameters (e.g., frame lengths), data can be reconstructed using Siemens e7tools software, or using open-source reconstruction methods such as STIR^26^. For researchers interested in using reconstructed data, we have additionally provided PET data reconstructed into 1-min bins using the OP-OSEM reconstruction software. The reconstructed image data is useful for developing post-processing pipelines^27^, and for exploring physiological and neuroscientific questions about glucose uptake in the brain^12,14^. Thus, the Monash vis-fPET-fMRI dataset is a uniquely powerful and flexible resource for the neuroimaging community.

In this data release, MR images, unreconstructed list-mode PET data and reconstructed PET images were converted to BIDS format prior to upload. While the BIDS specification for MR is mature, the BIDS specification for PET is still under development (https://github.com/ohbm/osr2020/issues/57)^28^, and does not include specifications for standardisation of list-mode data. In fact, the definition of ‘raw’ data for BIDS does not currently appear to extend to list-mode data; rather, the term refers to unprocessed reconstructed data (https://bids-specification.readthedocs.io/en/stable/02-common-principles.html#source-vs-raw-vs-derived-data). As such, we provide these currently unspecified data types using BIDS-style naming conventions and structures. Future BIDS releases may standardise structures and naming conventions for these currently unspecified data types, at which time we will endeavour to update the OpenNeuro data repository to reflect these new standards.

Simultaneous PET-MRI data is costly and difficult to acquire, requiring access to facilities that a small (but growing) number of biomedical imaging facilities worldwide currently possess. It is expected that release of this data in both raw and reconstructed formats will provide opportunities for further work validating synergistic multimodal reconstruction routines, algorithms for data preparation and signal detection optimisation; as well as new fundamental discoveries on the dynamic use of energy in the human brain.

## Data Availability

Scripts used to insert required metadata into the published BIDS dataset are freely available at https://github.com/BioMedAnalysis/petmr-bids under Apache License 2.0.

## Online-only Table

**Online-only Table 1:** Data fields available for the Monash visfPET-fMRI dataset.

Field	Description	Values	Notes
participants.tsv	Demographics and anthropometry data		
age	age of the participant	years	
sex	sex of the participant as reported by the participant	“M”: “male”, “F”: “female”	
handedness	handedness of the participant as reported by the participant	“L”: “left”, “R”: “right”	Self-report
education_years	years of education calculated as formal education of 6 months or more. starts from the first year of primary/elementary school onwards.	years	Note that Australian education system includes 13 years of school: 7 years primary/elementary 6 years high school.
education_specify	the highest level of formal education completed (i.e., does not include uncompleted education or education currently undertaken).	string	In Australia, ‘technical school’ is education undertaken in the TAFE/vocational education sector
efl	english as first language	“yes”: “Yes”, “no”: “No”	
visual_impairment	any kind of vision impairment including wearing reading glasses	“yes”: “Yes”, “no”: “No”	Self-report
visual_impairment_specify	description of visual impairment	string	Specified only for those responding ‘yes; to visual_impairment
mental_illness_history	self-report of whether the person has had a current or past Axis I psychiatric condition	“yes”: “Yes”, “no”: “No”	
mental_illness_specify	diagnosis or treatment of whether the person has received a diagnosis for any Axis I psychiatric condition	string	
mental_illness_family_history	self-report of whether the person has a family member who has had a current or past Axis I psychiatric condition	“yes”: “Yes”, “no”: “No”	
mental_illness_family_history_specify	diagnosis or treatment of the family member’s psychiatric condition	string	
dementia_suspec_dx_family	Whether a family member had suspected or diagnosed dementia	string	
neurological	Whether the person has a current or previous neurological condition	string	
cardiovascular	Whether the person has a current or previous cardiovascular condition	string	
diabetes	Whether the person has a current or previous diabetes diagnosis	string	
regular_medication	Whether the person is currently taking regular medication	“yes”: “Yes”, “no”: “No”	
regular_medication_specify	description of regular medication	string	
medication_last24hr	Whether the person took medication in the previous 24hrs	string	
current_smoker	currently smoking	“yes”: “Yes”, “no”: “No”	
previous_smoker	previous smoking	“yes”: “Yes”, “no”: “No”	
previous_smoker_specify	description of previous smoking	string	
alcohol_drinking_days	number of days per week/month the person drinks	numeric	If a person drinks less than once per week, a fraction is provided – e.g., 1 day in 3 weeks = 1/21
alcohol_number_drinks	number average number of standard drinks per drinking day	numeric	
recreational_drugs	used recreational drugs in last 6 months	“yes”: “Yes”, “no”: “No”	
recreational_drugs_specify	description of recreational drugs used in last 6 months	string	
edinbugh_hand_l	Edinburgh Handedness Inventory: left-hand	numeric	Total score for left handed items
edinburgh_hand_r	Edinburgh Handedness Inventory: right-hand	numeric	Total score for right handed items
cesd_r	Centre for Epidemiological Studies Depression Inventory - Revised total score	numeric	
dose	FDG dose in MBq	numeric	
bsl	Blood sugar level	numeric	
plasma_radioactivity.tsv	Plasma radioactivity data for each subject		
Infusion_start_time	Time infusion started	hr:min:sec	
T*_SampleTime	Time sample was taken		Multiple entries per timepoint, 0–10
T*_MeasurementTime	Time measurement of radioactivity was taken	hr:min:sec	Multiple entries per timepoint, 0–10
T*_CPM	Counts per minute for each timepoint	numeric	Multiple entries per timepoint, 0–10
T*_TC	Total counts for each timepoint	numeric	Multiple entries per timepoint, 0–10
sub_*			
anat	T1 weighted image data		
dixon	Scans acquired with Dixon sequence		Multiple acquisitions are stored separately
fmap	Functional maps in magnitude and phase		
func	Functional MRI data		Sessions are stored separately, e.g. *_fullchecker, *_half_checker, and *_rest
pet	Reconstructed PET images with 1-minute binning		Blood information is stored in *-blood_discrete.tsv
ute	Scans acquired with UTE sequence		Multiple acquisitions are stored separately.
